# Alcohol Content of Proprietary Medicines

**Published:** 1916-01

**Authors:** 


**Alcohol Content of Proprietary Medicines.** Unless the examination of the subject published in N. A. R. D. Journal, Nov. 4, is at fault, the alarm frequently expressed at the spread of the alcohol habit through proprietary medicines, has little foundation in fact. The revenue list of June 6, 1914, contained the names of 287 such preparations. Two of the largest wholesale price lists of proprietary medicines contained only 14 of these titles. Examination of several drug stocks and inquiries of several wholesale druggists, showed that practically none of these were sold to druggists, though they may be in use by saloons, or locally and privately to evade prohibitory laws. Of 1108 proprietary preparations, 308 or 27.79%, contained alcohol to the extent of 1% or more, but it is implied that none of these were adapted to use as beverages. In comparison, it is interesting to note that of 427 official Galenicals, 206 or 48.24% were alcoholic, and that of 575 N. F. Galenicals, 274 or 47.65% were alcoholic.

